# Statistical Coupling Analysis Predicts Correlated Motions in Dihydrofolate Reductase

**DOI:** 10.1021/acs.jpcb.4c04195

**Published:** 2024-10-10

**Authors:** Thomas L. Kalmer, Christine Mae F. Ancajas, Cameron I. Cohen, Jade M. McDaniel, Abiodun S. Oyedele, Hannah L. Thirman, Allison S. Walker

**Affiliations:** † Department of Chemistry, Vanderbilt University, Nashville, Tennessee 37240-0002, United States; ‡ Department of Biological Sciences, Vanderbilt University, Nashville, Tennessee 37240-0002, United States; § Center for Structural Biology, Vanderbilt University, Nashville, Tennessee 37240-7917, United States; ∥ Department of Cell and Developmental Biology, Vanderbilt University, Nashville, Tennessee 37240-7935, United States; ⊥ Department of Pathology, Microbiology and Immunology, Vanderbilt University Medical Center, Nashville, Tennessee 37232, United States; # Vanderbilt Center for Immunobiology, Vanderbilt University Medical Center, Nashville, Tennessee 37232, United States; ∇ Chemical & Physical Biology Program, Vanderbilt University, Nashville, Tennessee 37232-0301, United States; ○ Evolutionary Studies Initiative, Vanderbilt University, Nashville, Tennessee 37240-0002, United States

## Abstract

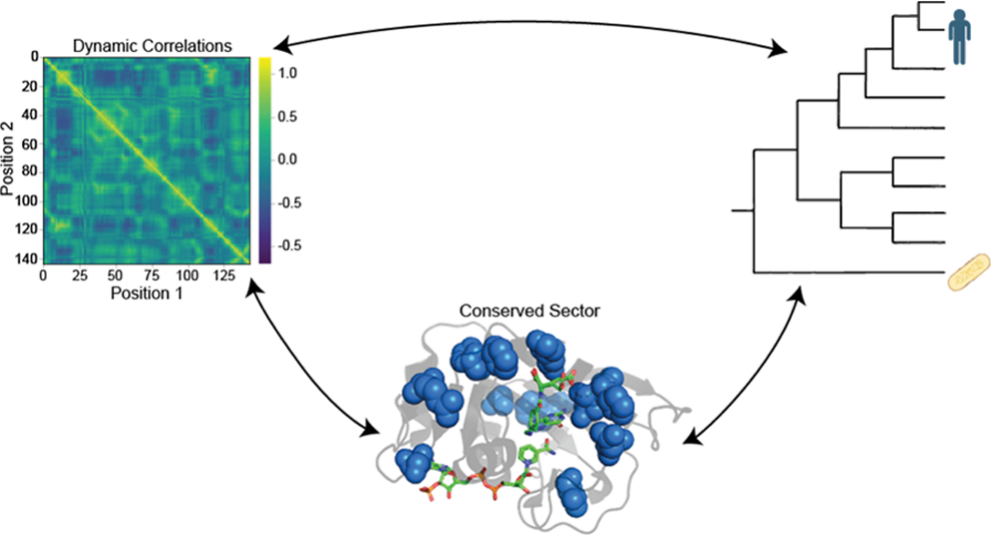

Dihydrofolate reductase (DHFR), due to its universality and the depth with which it has been studied, is a model system in the study of protein dynamics. Myriad previous works have identified networks of residues in positions near to and remote from the active site that are involved in the dynamics. For example, specific mutations on the Met20 loop in *Escherichia coli* DHFR (N23PP/S148A) are known to disrupt millisecond-time scale motions as well as reduce catalytic activity. However, how and if networks of dynamically coupled residues influence the evolution of DHFR is still an unanswered question. In this study, we first identify, by statistical coupling analysis and molecular dynamic simulations, a network of coevolving residues that possesses increased correlated motions. We then go on to show that allosteric communication in this network is knocked down in N23PP/S148A mutant *E. coli* DHFR. We also identify two sites in the human DHFR sector which may accommodate the Met20 loop double proline motif. Finally, we demonstrate a concerted evolutionary change in the human DHFR allosteric networks, which maintains dynamic communication. These findings strongly implicate protein dynamics as a driving force for evolution.

## Introduction

Enzymes play a crucial role in nearly all biological processes, catalyzing reactions that would otherwise be inaccessible in nature. The mechanism of these enzymes can vary greatly, but they all function to accelerate chemical reactions by lowering the activation energy. In this pursuit, enzymes move in a variety of ways to form the interactions necessary for catalysis. However, the field has yet to come to a consensus about just how much these movements, both large and small, contribute to the catalytic power of enzymes.^1−4^ Many publications have covered this debate, with several suggesting that much of the controversy is caused by the lack of clarity in important definitions, such as what time scale motions should occur in, how to define contribution to enzymatic power, and what experiments should be performed in order to prove significance.^1,2,4^

With the introduction and expansion of molecular dynamics (MD) simulations, along with other movement-encompassing techniques, an increasing number of results from biochemical literature suggest that dynamical motions, fundamental for catalytic power or not, are involved in a wide variety of enzymatic functions.^4−6^ These studies provide crucial insights into their respective systems. Moreover, since reaction rate differences of just 1 order of magnitude can easily impact survival, very small differences in enzymatic function as a consequence of hindered dynamics can impose considerable evolutionary pressure on biological systems.^2^ For this reason, it is logical to think that evolution may act to conserve specific amino acids or networks of amino acids as a mechanism for preserving important motions.

A model system to study the role of protein dynamics is dihydrofolate reductase (DHFR). DHFR is an enzyme responsible for the reduction of dihydrofolate to tetrahydrofolate in which the cofactor nicotinamide adenine dinucleotide phosphate (NADPH) acts as a hydride donor.^7−10^ Tetrahydrofolate and derivatives are essential for thymidylate and purine synthesis.^11,12^ Subsequently, inhibition of DHFR results in a disruption of DNA replication and eventual cell death.^11,13^ Due to its critical role in cell health, DHFR has become an attractive drug target for multiple diseases.^14,15^ Many DHFR inhibitors are already available to treat a wide range of diseases, including fungal infections, parasites, cancer, arthritis, Crohn’s disease, and many other inflammatory conditions.^12,16,17^ DHFR is also highly structurally conserved across the tree of life, although sequence homology is weak.^10,18−20^ Multiple studies have been conducted on the kinetics and conformational changes of DHFR, especially in *Escherichia coli* (*ec*DHFR).^7,8,21^ DHFR relies on a series of ligand-induced conformational changes to facilitate catalysis which in humans (*h*DHFR) occurs through a hinge opening motion of the enzyme.^5,19,22−24^ The enzyme exists in the hinge-open conformation when empty and then changes to the hinge-closed conformation upon ligand binding. The latter conformation tightly packs the active site and favors hydride transfer from NADPH. Meanwhile, the Met20 loop in *ec*DHFR is more flexible and adapts distinct occluded and closed conformations throughout the enzymatic cycle.^19^ Notably, though the human and *ec*DHFR undergo the same catalytic reaction, they differ in their catalytic efficiency and dynamic behavior.^18^ Despite these extensive studies on DHFR, further studies are required to assess the contribution of dynamics to catalysis and the role of evolution.^4−6^

Dynamic coupling in proteins is a mechanism in which two sites are dynamically linked, enabling long-range communication and cooperative interactions despite not necessarily being in direct physical contact.^25^ This mechanism is a crucial aspect of allosteric regulation, whereby ligand binding or mutation-induced conformational changes trigger responses in remote regions of the protein.^26^ The study of protein dynamics provides an understanding of biological processes at a molecular level, including signal transmission, protein interactions, disordered protein behavior, and nucleic acid movements.^6,27^ One example is a study of the protein U1A and RNA recognition process, which identified cooperative effects from an MD simulation and cross-correlations of atomic fluctuations calculation.^28^ Furthermore, these correlations were found to be in agreement with the results from a positional covariance analysis of several RNA recognition motif sequences. This study provided an initial insight into protein-RNA recognition via direct interaction and long-range communication through pairwise interactions.

Statistical coupling analysis (SCA), on the other hand, is a computational method that has been developed to analyze multiple sequence alignments (MSAs) of protein families in order to identify groups of coevolving residues that are referred to as “sectors.”^29,30^ These sectors represent spatially organized networks within protein structures that often connect positions in the active site to surface sites distributed throughout the protein.^25^ The application of SCA serves as a valuable tool for researchers to identify functionally critical sectors within proteins and shed light on the propagation and dissipation of perturbations within protein structures.^31^ SCA has been instrumental in identifying networks of coevolved amino acids in proteins, such as in the MutS DNA mismatch repair protein, explaining the allosteric regulation and protein dynamics.^31−33^ This method allows for the quantitative examination of the long-term correlated evolution of amino acids within protein families, highlighting the statistical signature of functional constraints arising from conserved communication between positions.^34^

While SCA has previously been used to successfully identify allosteric networks within a variety of proteins,^35−40^ the physicochemical interactions that enable communication through these allosteric networks and how evolutionary constraints are defined by these interactions are still poorly understood.^41−43^ The few studies that have examined how SCA sectors relate to dynamics have mostly focused on using SCA to predict which mutations will impact dynamics^32^ or combining SCA and molecular dynamics simulations to identify coupled positions^33^ or allosteric pockets for drug design.^44^ There have been relatively few studies that investigated how coevolving residues identified by SCA relate to dynamic networks within proteins, although the theoretical underpinnings of SCA and molecular evolution suggest that dynamic networks that are important for protein function should be represented as sectors. One study did find a strong overlap between sectors identified by a decomposition of a covariance matrix of structural dynamics and the sectors identified by SCA.^33^ However, this study was limited to a PDZ domain, which primarily serves as an anchoring domain and does not have enzymatic activity. Therefore, it is unclear whether the same relationship between SCA sectors and dynamic networks would be present in enzymes such as DHFR. Further investigation is required to understand the relationship between evolutionary and dynamic networks.

Here, we investigate how correlated motions in DHFR relate to coevolution of residues. To assess this, we utilized SCA and ran MD simulations on wild-type *ec*DHFR. SCA identified sectors and independent components (IC) containing coevolving residues throughout the enzyme, and pairwise dynamic cross-correlation (DCC) was used to detect the dynamic motions. We further analyze how perturbations of residues interacting in these SCA-identified networks affect dynamics by performing simulations on a previously designed N23PP/S148A mutant *ec*DHFR. Our findings illustrated decreased dynamics in the mutant *ec*DHFR. Furthermore, we discussed how evolutionary constraints may relate to protein dynamics and identified specific changes in the human DHFR sector relative to *E. coli*.

## Methods

### Statistical Coupling Analysis (SCA)

A representative protein alignment of 4422 sequences with 802 positions of DHFR was obtained from PFAM (PF00186) by using the Stockholm 1.0 format. Statistical coupling analysis was performed on the sequence alignment using the Python package pySCA6.0.^38^ After processing, the final alignment size was 3664 sequences with 146 positions, and following calculation of sequence weights, there were 3216 effective sequences. The scaSectorID script identified four groups of coevolving residues or independent components (IC) in *ec*DHFR. After fitting to an empirical statistical distribution, a cutoff of *p* = 0.95 was used to determine which positions significantly contributed to each IC. The full pySCA package is available on the Ranganathan Lab GitHub (https://github.com/ranganathanlab). Additionally, residues in each IC were ordered numerically, and an SCA by IC matrix was constructed. Code available on GitHub (https://github.com/Kalmertl/SCA-Predicts-Correlated-Motions.git).

### Molecular Dynamics (MD) Simulation

The MD simulations were conducted using AmberTools22 and Amber22 suites.^45,46^ Protein Data Bank^47^ entries 3QL3 and 3QL0 were used as the initial structures for the wild-type and mutant *ec*DHFRs, respectively, while 4M6K was used for the human DHFR.^48^ Nicotinamide adenine dinucleotide phosphate (NAP) cofactor and folate (FOL) ligand geometry were extracted from the appropriate PDB entry. FOL was protonated in GaussView and parametrized with Antechamber with the following options “-c bcc -nc -2 -at gaff2,” and the NAP was protonated using reduce in LEaP, geometry optimized, and energy minimized with Gaussian version 16 using the UFF molecular mechanics force field and parametrized with Antechamber with the following options “-c bcc -nc -3 -at gaff2 -ek scfconv = 1.d-8 ndiis_attempts = 1000 grms_tol = 0.002.” H++^49^ was used to obtain the correct protonation states of the amino acids throughout the structures at a pH of 6.5. The program *xleap*(45) was used to apply ff19SB^50^ and GAFF2^51^ force fields to the proteins and ligands. The models were neutralized with Na^+^ ions and solvated by using the SPC/E water model in a truncated octahedral box with a buffer of 14 Å. Before the MD simulation, a process of minimization, heating, and equilibration was performed. Steepest descent minimization of the solvated system with a restraining force of 500.0 kcal/mol Å^–2^ on the protein was initiated in 500 steps, followed by 500 steps of conjugate gradient minimization. This process was iterated for the entire system at 0.0 kcal/mol Å^–2^ with 1000 steps of steepest descent minimization, followed by another 1500 steps of conjugate gradient minimization. Subsequent equilibration and heating of the system from 0 to 300 K using the Langevin temperature scheme and a 10.0 kcal/mol Å^–2^ constraint on the protein and ligands for over 20 ps simulation were performed. Final equilibration at a constant pressure of 1 atm and constant temperature at 300 K for 100 ps was conducted to relax the system to an equilibrium density. Explicit solvent MD simulation was continued at constant pressure for each DHFR protein for 30 ns, and the time step was set to 2 fs with the trajectory snapshots saved at every 5 ps. The *cpptraj* package^52^ included in AmberTools22 was used to compute the dynamic cross-correlation (DCC) matrix and root-mean-squared deviations (RMSD). Molecular visualization was carried out using PyMOL.^53^

### DCC Comparison with SCA

Pairwise RMSD values from the DCC matrix were mapped according to the IC assignment. Any amino acid pairs within 5 Å or two positions of each other were excluded. Pairwise RMSD values where both residues fell within a given IC were assigned to that IC for analysis. Further, pairwise values were assigned to “No IC” if neither amino acid in the pair belonged to an IC, “Any IC” if both amino acids belonged to an IC but not necessarily the same IC, and “not within same IC” for all possible pairs except those within the same IC. Using the absolute values for pairwise correlations, significant differences between distributions were determined via a two-sided Mann–Whitney *U* (MWU) test. DCC matrix residue numbering was shifted up by one after position 23 in the3QL0 mutant to account for the insertion (position 23 became residue 24 etc.). RMSD value extraction, IC assignment, and statistical analysis were performed by using a custom python script. GitHub available (https://github.com/Kalmertl/SCA-Predicts-Correlated-Motions.git).

## Results and Discussion

### SCA Identifies Four Independent Components in DHFR

**Figure 1:**
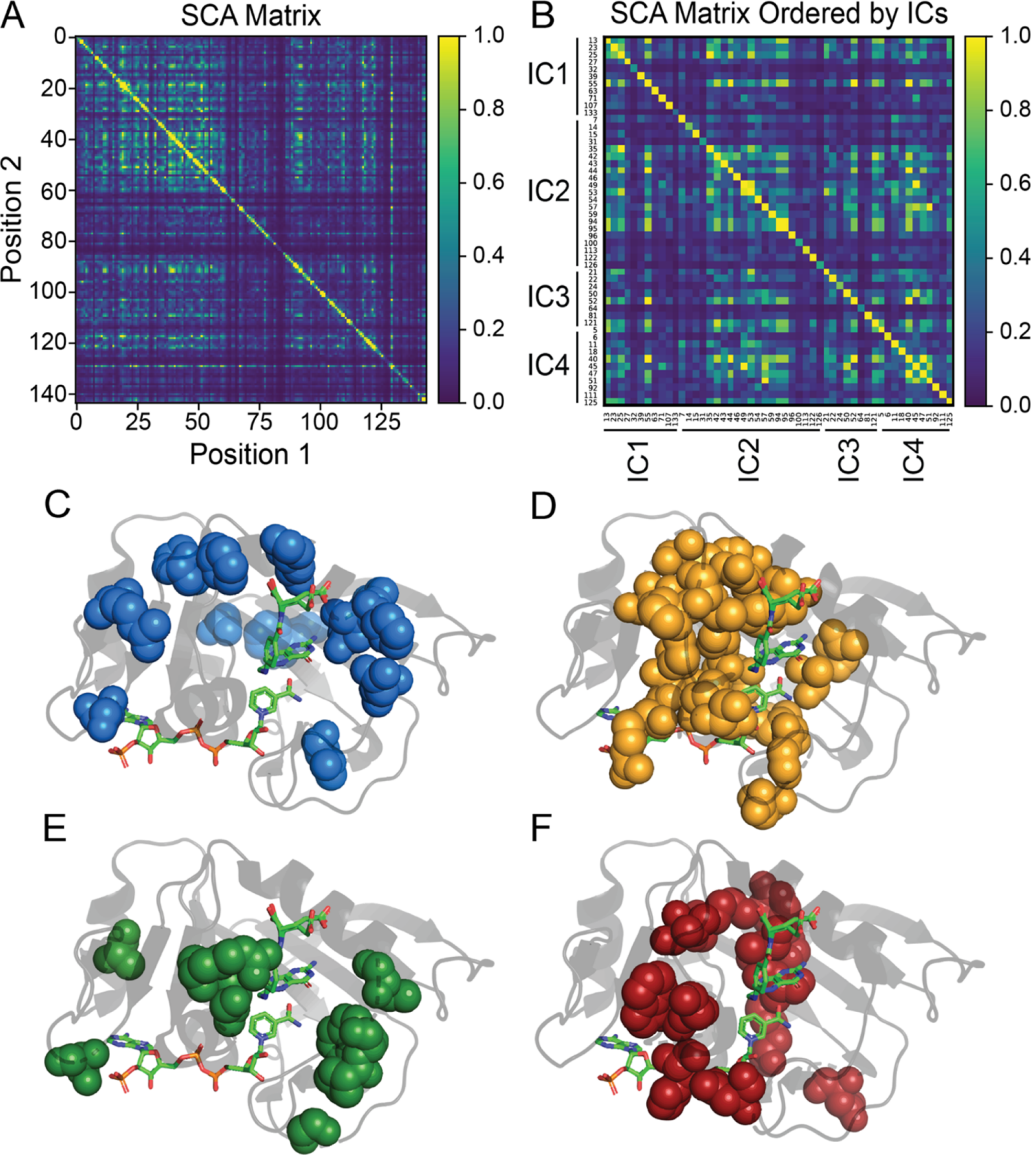
SCA matrix (A) constructed using the pySCA package as well as a custom matrix with IC contributors ordered numerically as (B). Additionally, the respective mapping of each independent component (IC1 - IC4) in DHFR, as determined by statistical coupling analysis, is shown in panels (C–F). The specific positions contributing to each IC can be found in panel (B).

To analyze the coevolutionarily conserved residues within DHFR, we applied statistical coupling analysis (SCA).^29^ By examining the SCA matrix (Figure 1A), we identified coevolving residues, called sectors, encompassing the active site, cofactor- and substrate-binding sites, and other distant positions that are consistent with previous studies.^25,34,54^ As noted by Reynolds et al., several residues within these sectors coincide with millisecond fluctuations involved in important dynamic motions underlying catalysis.^25,48,55,56^ Additionally, other studies have underscored the importance of coevolving residues captured by SCA, linking them to essential protein functions.

Through decomposition of the SCA matrix, we identified four coevolving independent components (ICs) within DHFR (Figure 1B and Table S1). Independent component analysis (ICA) is designed to maximize the statistical independence of the ICs. In many cases, ICs still show some dependence on each other, and these ICs can be combined into larger sectors.^38^ We observed the dependence between ICs in DHFR (Figure 1B) but chose to analyze each IC separately to provide better insight into how ICs relate to protein dynamics. Mapping each of the ICs onto DHFR revealed ICs clustered around the active site as well as distantly positioned surface sites. IC1 and IC3 (Figure 1C,E) are spatially noncontiguous, while IC2 and IC4 (Figure 1D,F) demonstrated a high degree of physical connectivity, localizing mainly to the cofactor and substrate-binding sites of DHFR. Previous studies have underscored the significant role many of the residues within these ICs play with regard to protein function and dynamics. Specifically, Asp27, found in the active site and captured by IC1, is known to coordinate with the pterin ring of folate.^57^ One functionally significant region within DHFR is the Met20 loop (residues 9–24), which changes conformation throughout the catalytic cycle of DHFR from the closed (E:NADPH and E:NADPH:DHF) to the occluded (E:NADP+:THF, E:THF, and E:NADPH:THF) states.^56^ Two other important loops are F-G (residues 116–132) and G-H (residues 142–150), providing stability with the Met20 loop.^10^ Several of these highlighted residues are captured within the four ICs. For example, Pro21, Trp22, and Asn23 that are involved in Met20 hinge motion are within IC3. IC4 also contains Tyr100 and Phe125, which were previously identified to play a key electrostatic role during hydride transfer, while a theoretical mutagenesis study has also implicated Asp122 within IC2 to affect coupled functional motions.^57−61^ By further analyzing the SCA matrix, we begin to unravel the interactions within these networks of residues in the ICs. Whether these residues are near or distant from important sites in DHFR, their interactions suggest further implications for allosteric regulation and other functional dynamics.

### Molecular Dynamics Simulations of DHFR Reveal Correlated Motions Within ICs

**Figure 2:**
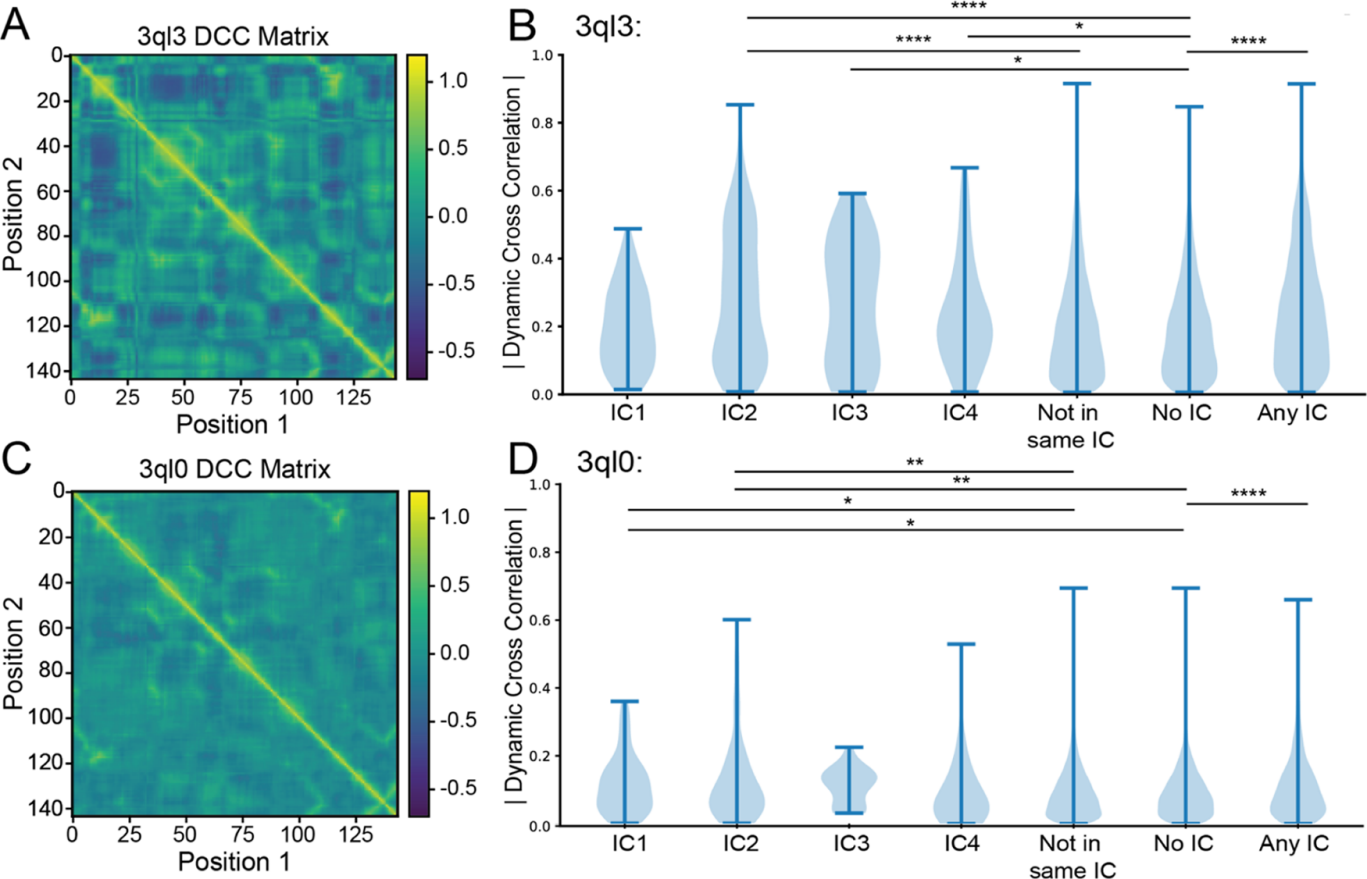
Dynamic cross-correlation matrix for wild-type (A) and mutant (C) *E. coli* DHFR (PDB: 3QL3 and 3QL0, respectively) in the E:NADP+:FOL state and the respective absolute value breakdown of their pairwise motions by independent component (B, D). Categories “Not in Same IC,” “No IC,” and “Any IC” correspond to all pairs of amino acids that lie outside of a given independent component, pairs of amino acids where neither amino acid has an independent component assignment, and all pairs where each amino acid is assigned to an independent component, respectively. Significance was determined using a two-sided Mann–Whitney *U* test and labeled following the convention *p ≤ 0.05, **p ≤ 0.01, ***p ≤ 0.001, and ****p ≤ 0.0001.

Previous studies have shown that SCA sectors overlap in DHFR with residues experimentally determined to be involved in millisecond motions as well as surface residues that allosterically regulate the active site.^25,48,55,56^ These sites include residues in the Met20 loop. However, there has been little investigation into the mechanisms underlying allosteric communication in sectors. One possibility is that the motions of positions in the same IC or sector are coupled. To determine how well SCA can predict correlated motions within a protein, we ran a 30 ns MD simulation on wild-type *E. coli* DHFR (starting structure PDB: 3QL3) (Figures S1 and S2). After extracting the pairwise dynamic cross-correlations and constructing a matrix, we were able to determine whether dynamic motions within each IC significantly varied from the rest of the proteins (Figure 2A,B). Because the ICs are relatively small (8 residues in the case of IC3) and often contain sequential amino acids which can skew correlated motions, the pairwise correlations for residues within 5 Å or 2 positions of each other in the sequence were not considered (i.e., the correlation coefficient between residues 50 and 52 in IC3 was not included in the analysis).

Using a Mann–Whitney *U* test, it was determined that the correlated motion distributions for IC2 were significantly different from those that were measured for residue pairs partially or fully outside of a given IC (“not in same IC”) (*p* = 1.30e–07). Furthermore, for those where both residues did not belong to an IC (“No IC”), IC2 showed a high degree of significant difference (*p* = 1.80e–08) (Figure 2B). IC3 and IC4, likely due to their small size, showed moderate significance when compared with the “No IC” assignment but not with “Not in same IC” (*p* = 0.0480 and *p* = 0.0708 for IC3 and *p* = 0.045 and *p* = 0.0772 for IC4, respectively) (Table S2). Additionally, the mean correlated motion of IC3 is higher than that of the “No IC” as well as “Not in same IC” categories (Table 1). IC1 showed no significant increase in the correlated motions. This is in line with the 2015 findings by Teşileanu et al. that the top eigenmode, the mode which represents the most variance, of the SCA matrix may be independent of evolutionary correlations between positions and instead may capture general patterns of conservation.^30^

**Table 1:** Average Values for Dynamic Correlations in Wild-Type (3QL3) and Mutant (3QL0) MD Simulations Broken Down by SCA Assignments

category	3QL3 average value	3QL0 average value
IC1	0.18429688	0.11890625
IC2	0.26761798	0.12969663
IC3	0.25324	0.10978947
IC4	0.22229412	0.09768627
not in same IC	0.18869158	0.09741831
No IC	0.18217110	0.09667329
Any IC	0.23073404	0.11503578

Interestingly, the distribution of pairwise correlations for all residues assigned to an IC (“Any IC”) was significantly different when compared with “No IC” (*p* = 3.46e–18). Furthermore, the individual top pairwise correlations, Gly15-Trp47 and Gly15-Gly121, while captured by SCA, were not captured within any of the ICs individually and rather occurred across multiple ICs. Gly15 belongs to IC2 and Trp47 to IC4, while Gly121 belongs to IC3. This point illustrates the benefit of grouping ICs into sectors, especially when considering protein dynamics, and further strengthens the notion that the top eigenmode represents general evolutionary patterns. Finally, as is apparent by the range of the “No IC” category in Figure 2B, evolutionarily conserved positions do not capture all highly correlated motions. Regardless of misgrouping top contributors and failing to completely capture all coupled motions, SCA enriched for a disproportionate amount of correlated motions and did so while capturing multiple residues that have been experimentally determined to be catalytically and dynamically important.

### Mutated DHFR Shows Dampened Motions Within ICs

In 2011, the Wright group published a double mutant *E. coli* DHFR, N23PP/S148A, which destabilized the occluded form of the Met20 loop, disrupted millisecond-time scale motions within the active site, and severely reduced catalytic activity.^48^ The authors also created single mutant proteins S148A and N23PP. The S148A mutant trapped the Met20 loop in the closed position but retained millisecond motions in active site residues, while the N23PP mutant abrogated active site residue motions. Ser148, located C-terminally from the G-H loop, forms a hydrogen bond with Asn23 that lies directly within the Met20 loop. Disruption of this bond can directly account for the abrogation of the Met20 loop conformational changes observed in the generated mutant. However, the mechanism by which the N23PP/S148A double mutant abrogates other active site residue dynamics remains unaccounted for.

To investigate whether disrupting allosteric communication in the ICs of *ec*DHFR can explain the unaccounted-for dynamic change, we ran a 30 ns MD simulation on a double mutant (starting structure PDB: 3QL0) (Figures S1 and S2). Consistent with the Wright group’s findings, our MD simulation displayed dramatically decreased correlated dynamics relative to the wild type. Visual comparison of the dynamic cross-correlation matrices in Figure 2A,C showcases the decreased correlations. After breaking down the dynamic cross-correlation matrix by SCA assignment and excluding amino acid pairs within 5 Å or 2 positions of one another, as was done for the wild type, we were able to inspect the behavior of evolutionarily conserved networks in the abrogated mutant. Notably, when using a two-sided Mann–Whitney *U* test, IC4 lost significance when compared with the “No IC” category (*p* = 0.219). Similarly, IC2 in mutant *ec*DHFR lost multiple orders of significance when compared with “No IC” (*p* = 0.00922 for mutant and *p* = 1.80e–08 for wild type). Furthermore, when examining the averages for “No IC” and “Any IC” (two-sided *p* = 9.42e–05) in wild-type and mutant *ec*DHFR, it is clear that the mutant distributions are closer than the wild type (Tables 1 and S2).

To further probe the differences between wild-type and mutant dynamics, we performed a statistical analysis. Most prominent was the shift for IC2 (*p* = 6.17574e–12) and IC4 (*p* = 2.43828e–06). While IC1 and IC3 distributions showed a significant change, the significance was markedly less (*p* = 0.00283 and *p* = 0.03087, respectively) (Table S3). Again, these findings mirror the initial description of mutant dynamics by the Wright group: the dynamics of amino acids around the active site loops are most highly perturbed. The shift for “No IC” and “Any IC” was also both highly significant (*p* = 7.79406e–193 and *p* = 5.31155e–78, respectively) (Table S3). Overall, these trends indicate that dynamics in IC-associated positions, especially IC2 and IC4, were significantly decreased as a result of the N23PP/S148A mutant.

**Figure 3:**
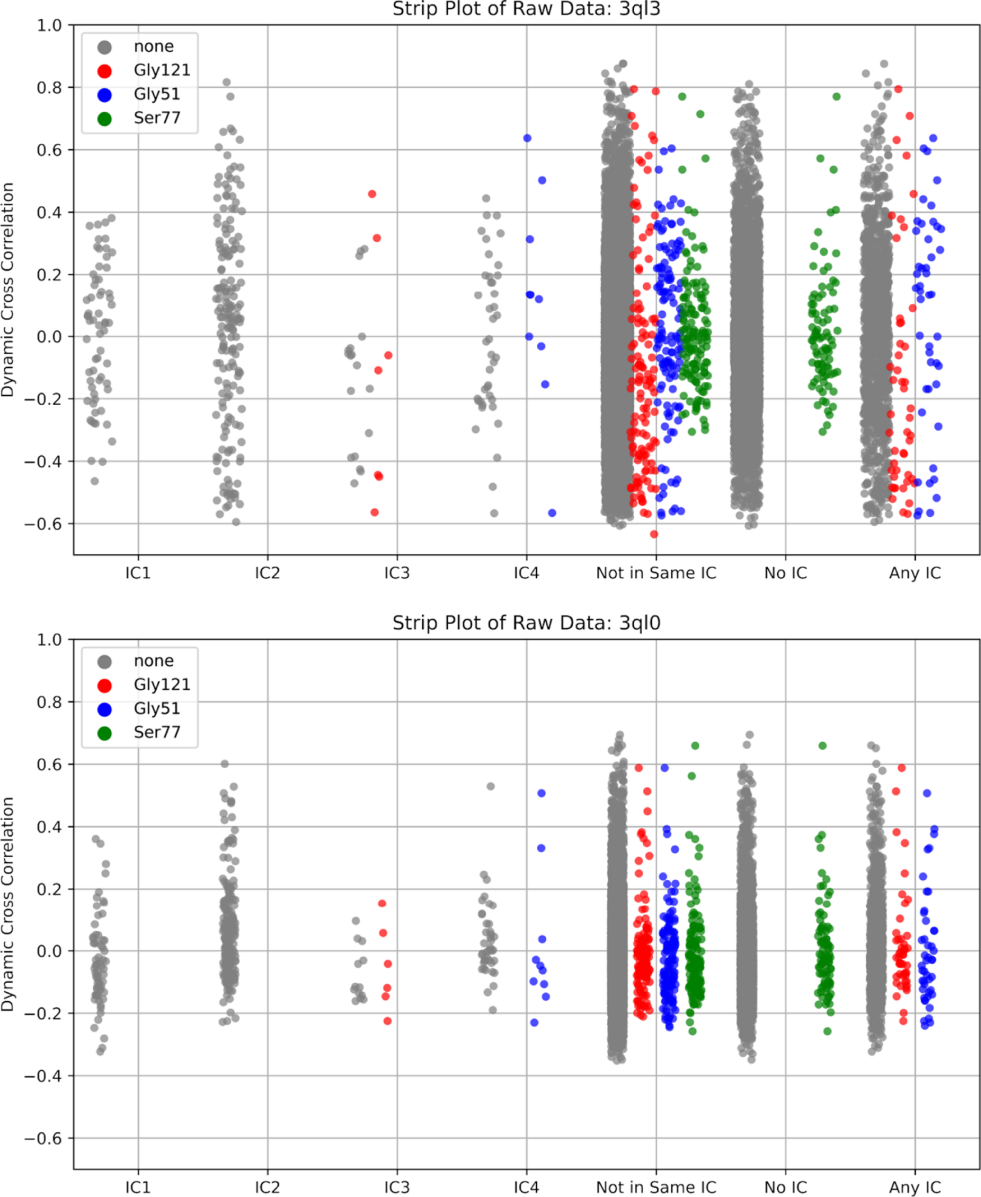
Raw pairwise dynamic cross-correlation data for wild-type (3QL3, top) and mutant *ec*DHFR (3QL0, bottom). Pairwise correlations involving the residues Gly121, Gly51, and Ser77, respectively labeled red, blue, and green, were highlighted for their known millisecond conformational exchange as determined by CPMG and their respective involvement in active site loop conformation, substrate/product binding, and cofactor binding.

Admittedly, the extent to which our nanosecond time scale MD simulation was able to recapitulate the full scale of dynamic changes in the N23PP/S148A double mutant, which were originally evaluated on the millisecond-time scale by Carr–Purcell–Meiboom–Gill (CPMG)–based R2 relaxation dispersion experiments, may be limited.^48^ To probe how well our simulation was able to recapitulate the CPMG results of Michaelis model complex movements, we examined the behavior of three amino acids: Gly121, Gly57, and Ser77. At various points throughout the catalytic cycle, these residues displayed millisecond-time scale movements in active site loop conformations, substrate/product binding, and cofactor binding, respectively.^55^ As expected for the Michaelis model complex, we observed the most dramatic changes in active site loop conformation movements (Gly121) while the substrate/product (Gly57) and cofactor binding (Ser77) changes were less pronounced, (Figure 3). Taken together, these results suggest that dampening of an evolutionarily conserved allosteric network within *ec*DHFR, as identified by SCA, serves as the mechanistic link by which the N23PP/S148A double mutation produces a dramatic knockdown of active site dynamics in *ec*DHFR.

### Human DHFR Sector Shows Two Compensatory Mutations

**Figure 4:**
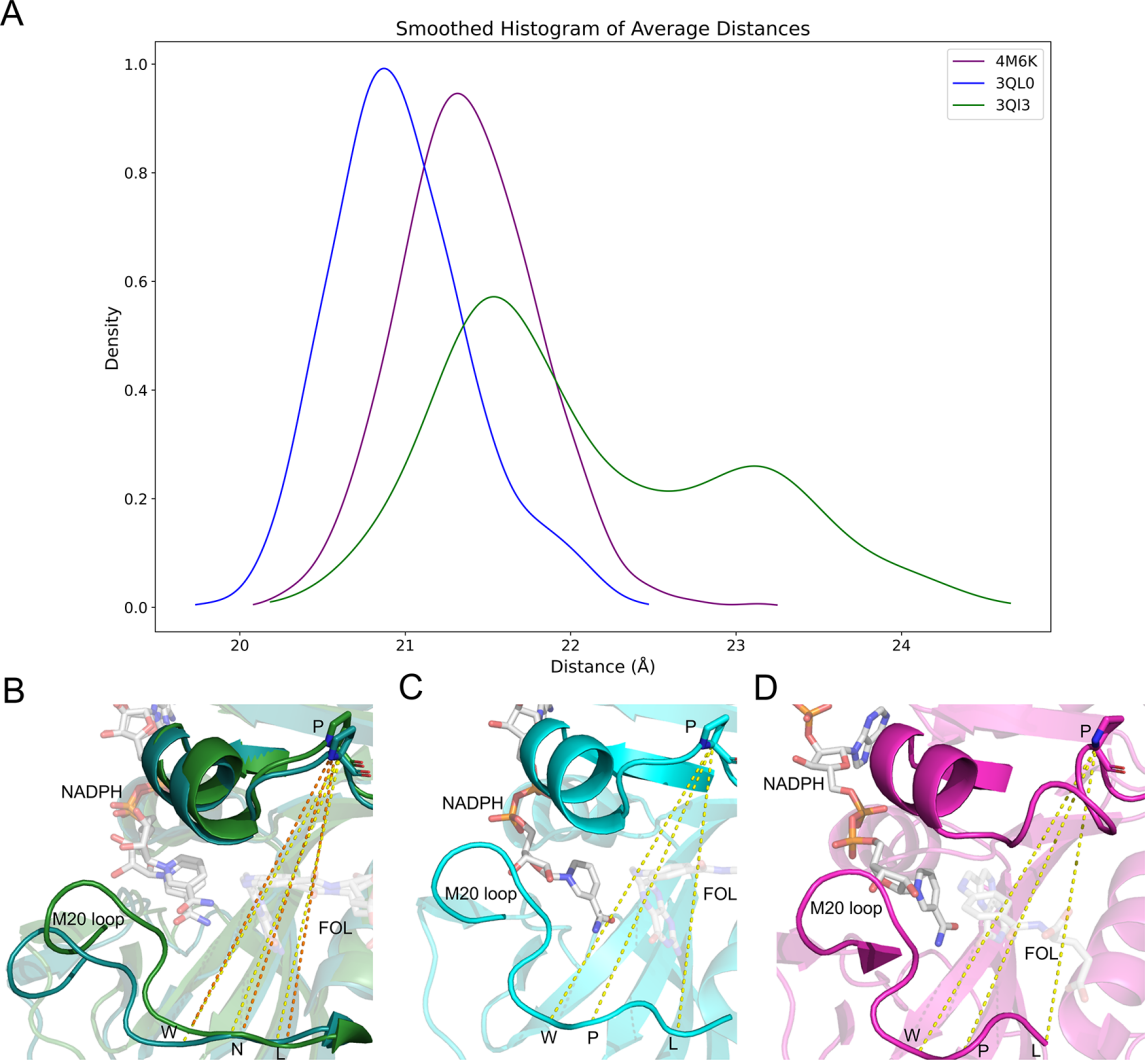
Average hinge distances (Å) for wild-type *ec*DHFR (3QL3, green), mutant *ec*DHFR (3QL0, blue), and *h*DHFR (4M6K, purple). Distances were measured at positions W22–P53, N23–P53, and L24-P53 for the wild-type *ecDHFR* (depicting two representative structures during the simulation) (B) while mutant *ec*DHFR hinge distance is the average of the distance between positions W22–54, P23–54, and L25–54 (C). Human DHFR hinge distances were averaged between positions W24–P66, P25–P66, and L27-P66 (D). All measurements were taken at the α carbon and measured throughout the 30 ns simulation.

Recent evidence from a group led by Doeke Hekstra advanced the link between the Met20 loop dynamics and total *ec*DHFR dynamics. Specifically, the group identified a global hinge motion that is monotonically linked to Met20 loop backbone dihedrals. Interestingly, the authors note that their observed hinge motion is present and more pronounced in *h*DHFR upon product release despite the fact that *h*DHFR bears the N23PP motif.^62^ In line with the Hekstra group’s findings, our examination of N23PP/S148A mutant *ec*DHFR revealed decreased hinge motions relative to wild type (Figures 4A–C and S3). Interestingly, our 30 ns MD simulation of Michaelis complex *h*DHFR (pdb: 4M6K) (Figures S1 and S2) showed a larger average hinge distance than N23PP/S148A *ec*DHFR but a similar variance in hinge distance (0.179 and 0.180, respectively) (Figures 4A,D and S3). Our findings, along with the Hekstra group’s, raise the question of how the network of conserved amino acids identified in *ec*DHFR have evolved in human DHFR to retain the global hinge motion while simultaneously harboring the N23PP mutation.

To probe this question, we first mapped DHFR’s independent components onto *h*DHFR (PDB: 4M6K) using the same multisequence alignment from which we identified the ICs in *ec*DHFR (Table S4). Because we previously determined that the top eigenmode (IC1) was not dynamically relevant in *ec*DHFR, we decided to focus our analysis on ICs 2–4, hereafter termed a sector. After using PyMOL to view the *E. coli* and human sectors within their respective proteins and create a structure-based alignment (Figure S4A), we were able to examine changes to the conserved allosteric network. Two regions in the *h*DHFR sector do not structurally align well with the *E. coli* sector: *h*Pro23/*h*Trp24 and *h*Pro61/*h*Arg65 (Figure S4B). The misalignment at *h*Pro23 and *h*Trp24 relative to *ec*DHFR is unsurprising, as these are the two amino acids immediately preceding the Pro-Pro motif from which the N23PP *E. coli* mutation was derived. There is an insertion at the *h*Pro61/*h*Arg65 site relative to the contiguous corresponding positions in the *E. coli* sector (*e*Gly51 and *e*Arg52). Interestingly, the *h*61-PEKN-65 sequence has been previously identified to rescue N23PP *E. coli* mutant catalysis when inserted in place of *e*Gly51.^23^ While this catalytic recovery indicates the evolutionary benefit of the *h*61-PEKN-65 sequence in the presence of a Met20 loop which contains a double proline, we are unaware of any direct analysis on dynamics in a G51PEKN N23PP double mutant *ec*DHFR.

After exploring the structural misalignments in the *E. coli* and human sectors, we explored point mutations in the sector. Again, using the structural alignment, we identified four point mutations in the *h*DHFR sector: *h*Gly20, *h*Trp113, *h*Phe134, and *h*Ser144. The corresponding positions are *e*Asn18, *e*Met92, *e*Tyr100, and *e*Gly121, respectively. We parsed our original MSA of 4422 DHFR sequences to examine the co-occurrence of these point mutations with the Pro-Pro motif in the Met20 loop. In our MSA, 36 sequences possessed the Met20 Pro-Pro motif. Interestingly, we found that out of these 36 sequences, the only two instances in which the *h*Gly20 position was a nonglycine residue were the same two instances in which the position corresponding to *h*Pro61 was nonproline (Table S5). Moreover, glycine occurred in the *h*Gly20 position without the presence of the Met20 Pro-Pro motif in 1858 sequences, and proline occurred in the *h*Pro61 position without the Met20 Pro-Pro motif in 2207 sequences. This demonstrates that the *h*Gly20 and *h*Pro61 mutations do not significantly disrupt the function of DHFR in the absence of the Met20 Pro-Pro motif. Furthermore, these results, along with the previous creation of a catalytically active N23PP/G51PEKN *ec*DHFR mutant, strongly indicate that *h*Gly20 and *h*Pro61 mutations are important for maintaining DHFR function in the presence of the Met20 Pro-Pro motif and suggest an evolutionary pathway to sequences with a Met20 Pro-Pro motif.

**Figure 5:**
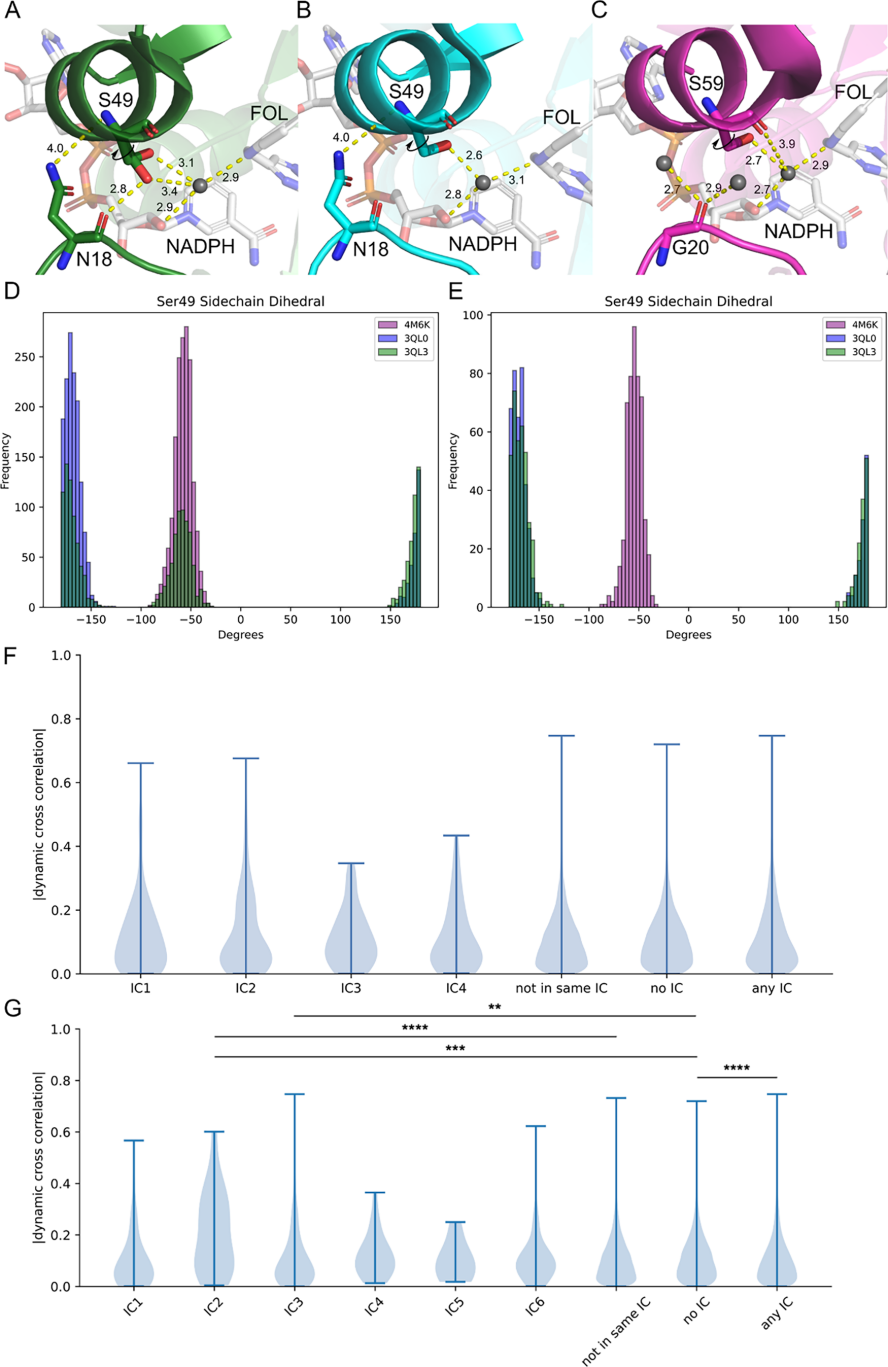
PDB structures for wild-type *ec*DHFR (A), N23PP/S148A mutant *ec*DHFR (B), and *h*DHFR (C) with key interactions (<5 Å) between the ligands, water molecules, and residues in the active site highlighted (3QL3, 3QL0, and 4M6K, respectively). Dihedral distribution for the Ser49 side chain of the *ec*DHFR and *h*DHFR assessed for MD simulation frames closely representing the open state (D) and the closed state (E) in the wild-type *ec*DHFR (See the Supporting Information). The absolute value of the pairwise motions by independent components for the human DHFR from the initial MSA (F) and from MSA with sequences >40% similarity to *h*DHFR (G). Categories “Not in Same IC,” “No IC,” and “Any IC” correspond to all pairs of amino acids that lie outside of a given independent component, pairs of amino acids where neither amino acid has an independent component assignment, and all pairs where each amino acid is in assigned to an independent component, respectively. Significance was determined using a two-sided Mann–Whitney *U* test and labeled following the convention *p ≤ 0.05, **p ≤ 0.01, ***p ≤ 0.001, and ****p ≤ 0.0001.

In order to contextualize these findings in our analysis of DHFR dynamics, we closely examined the structures of wild-type *ec*DHFR, N23PP/S148A *ec*DHFR, and *h*DHFR along with their associated electron densities (PDB codes 3QL3, 3QL0, and 4M6K, respectively). In doing so, we noted a dramatic shift in the side-chain rotamer distribution of *e*Ser49 between wild-type and mutant *ec*DHFR. The wild-type *e*Ser49 side chain dynamically communicates between the backbone carbonyl of *e*Asn18, which lies directly within the Met20 loop, and a water molecule (occupancy of 0.5 for each rotamer)^48,63,64^ (Figure 5A). In the mutant crystal structure, there is strong density for a single rotamer of *e*Ser49 that forms a hydrogen bond with the aforementioned water (Figure 5B). The communication between *e*Asn18 and *e*Ser49 is structurally reinforced by hydrogen bonds between the *e*Asn18 side chain and the α helix to which *e*Ser49 belongs. This led us to postulate that *e*Asn18 plays a role in the coupling of Met20 motions and the global hinge motion. To investigate further, we examined the *e*Ser49 side-chain rotamer distribution throughout various portions of the wild-type, mutant, and human DHFR simulations (Figure 5A–C). Because the wild-type *ec*DHFR simulation showed multiple conformational changes in the Met20 loop (Figure S5), we focused our analysis on the frames where *ec*DHFR was in the most open-like state (Figure 5D) and where the Met20 loop was the most closed-like (Figure 5E). Visualization of these conformations can be found in Figure S6. We observed that in the most open state, the wild-type *e*Ser49 side chain displayed a bimodal distribution at ±180 and −60° (Figure 5D). During the period when the Met20 loop was most closed, the wild-type *e*Ser49 rotamer resembles that of the dynamic knockout (Figure 5E). Similar to the dynamic knockout, the human serine rotamer (*h*Ser60) shows a single distribution throughout the simulation as well as no apparent contact with *h*Gly20 (Figure 5C–E). These findings indicate that conformational changes in the Met20 loop directly affect the dynamics of the *e*Ser49 rotamer. Interestingly, when we examined the variance in the hinge motion for wild-type *ec*DHFR at the most closed and most open states, we observed a dramatic shift. For the period when the Met20 loop was most open, the variance for the average hinge distance measured across all three sites was 0.2834. However, when wild-type *ec*DHFR was in the closed conformation, the variance decreased to 0.1178. Based on our analysis and the findings of the Hekstra group, we propose a model whereby the backbone dihedral angles of the Met20 loop in *ec*DHFR allosterically communicate across the active site cleft via *e*Asn18 in the closed state to affect the global hinge motion. To escape the deleterious effect that the double proline motif has on the Met20 loop dihedrals and, consequently, overall hinge motion, the human sector has partially decoupled Met20 loop dihedrals from global hinge motion by evolving a Glycine at the *e*Asn18 position.

Finally, we sought to directly determine whether the conserved amino acid networks in human DHFR have indeed evolved to accommodate the Met20 loop double proline motif and recover dynamic communication. As mentioned, we used our original MSA to identify four independent components in *h*DHFR with SCA. The dynamic correlations within these four independent components as well as for the sector representing the combination of these four ICs (Any IC) showed no significant increase over non-IC residues (No IC) (Figure 5F). However, given that the conserved network that we mapped onto *h*DHFR was derived using DHFR sequences from all domains of life and because we previously observed a dynamic knockdown throughout this same network in N23PP/S148A *ec*DHFR, these results were unsurprising. Originally, we sought to understand how conserved networks within human DHFR had changed relative to *E. coli*, but attempting to identify these networks through an MSA laden with bacterial DHFR sequences obfuscates this goal. To better address the question, we returned to the MSA processing steps of SCA and set the “min SID to reference seq” parameter to 0.4, with *h*DHFR as the reference. This had the effect of excluding all sequences with less than 40% sequence identity to *h*DHFR, biasing our analysis toward identifying conserved allosteric networks within eukaryotic DHFR. After completing SCA with our new MSA, we identified six independent components in *h*DHFR (Table S7). Parsing the *h*DHFR MD simulation into the new ICs revealed that IC2 and IC3 correlated dynamics were significantly higher than non-IC residues (*p* = 1.80e–04 and *p* = 0.00937, respectively) (Figure 5G). Interestingly, IC2 and IC3 are positioned on either side of the active site cleft (Figure S7). Furthermore, the “Any IC” category gained a significant difference from “No IC” (*p* = 5.91e–05). However, the average values for “Any IC” and “No IC” are 0.112 and 0.119, respectively, indicating that allosteric communication throughout the entire conserved network is not increased and rather only the residues in IC2 and IC3. Taken together, this evidence demonstrates a concerted evolutionary change in the architecture of the allosteric networks in human DHFR, which allows for dynamic communication in the presence of an otherwise deleterious Met20 loop double proline motif.

## Conclusions

This paper is the first example to demonstrate the ability of SCA to identify networks of coevolving residues in an enzyme that exhibit an increase in correlated motions relative to the remainder of the protein. We demonstrated that increased correlation in dynamics is apparent when viewing these networks from both the individual IC level as well as grouping ICs into a sector. Furthermore, we were able to show that the dynamic communication of this network is decreased by a naturally occurring double proline motif. Specifically, the relative changes of “Any IC,” IC2, IC3, and IC4 with respect to “No IC” in the mutant *ec*DHFR show a clear decrease in the dynamics of SCA-identified residues. Through the lens of SCA, we also demonstrated that conserved networks within DHFR have coevolved to accommodate an otherwise dynamically deleterious mutation present in human DHFR. We believe that our model of allosteric communication from the Met20 loop across *e*Asn18 to *e*Ser49 offers an explanation and a starting point for future experimentation as to how human DHFR has decoupled Met20 backbone dihedrals from the global hinge motion. More broadly, our findings implicate protein dynamics as a driving force for evolution. That is, our work has shown that electronic perturbations to networks of amino acids that exhibit correlated dynamics are more likely to be evolutionarily selected against. This concept will undoubtedly prove useful in the design and understanding of enzymes.

## Supporting Information Available

*E. coli* IC residue compositions; within variant *p*-values from Mann–Whitney *U* (MWU) tests of dynamic correlations; within and across variant *p*-values from MWU tests of dynamic correlations for *ec*DHFR; human DHFR IC residue compositions; proteins for which the Met20 Pro-Pro motif was present in original alignment and did not possess the Gly20 or the 61-PEKN-65 mutation; within variant *p*-values from MWU tests for the human DHFR; new *h*DHFR IC residue compositions from MSA of sequences with greater than 40% similarity to *h*DHFR; RMSD of wild-type and mutant DHFR with respect to initial structure; distance between folate ligand and active site residue of wild-type and mutant DHFR; distribution and average hinge distance for wild-type and mutant DHFR; overlaid PDB structure for human and *ec*DHFR; top representative structures throughout the simulation for wild-type ecDFHR, mutant *ec*DHFR, and *h*DHFR; conformations of the wild-type *ec*DHFR closely representing the more close- and open-like states during the simulation; and mapping of *h*DHFR IC2 and IC3 (PDF)
