# Supplementary material for: Statistical Coupling Analysis Predicts Correlated Motions in Dihydrofolate Reductase
Source: J Phys Chem B. 2024 Oct 10;128(42):10373–84. doi: 10.1021/acs.jpcb.4c04195 (PMC11514014; doi:10.1021/acs.jpcb.4c04195)
Supplement: Supplementary file 1 — jp4c04195_si_001.pdf [file jp4c04195_si_001.pdf]

Supporting Information: Statistical Coupling Analysis Predicts Correlated Motions in Dihydrofolate Reductase

Thomas L. Kalmer,*[1] Christine Mae F. Ancajas,*[1] Cameron I. Cohen,[2,3] Jade M. McDaniel,[2] Abiodun S. Oyedele,[1] Hannah L. Thirman,[4,5,6,7] Allison S. Walker[1,2,5,8 #]
* Authors contributed equally
# Corresponding author: allison.s.walker@vanderbilt.edu
1. Department of Chemistry, Vanderbilt University Nashville, TN, USA
2. Department of Biological Sciences, Vanderbilt University, Nashville, TN, USA
3. Center for Structural Biology, Vanderbilt University, Nashville, TN, USA
4. Department of Cell and Developmental Biology, Vanderbilt University, Nashville, TN, USA
5. Department of Pathology, Microbiology and Immunology, Vanderbilt University Medical Center, Nashville, TN, USA
6. Vanderbilt Center for Immunobiology, Vanderbilt University Medical Center, Nashville, TN, USA
7. Chemical & Physical Biology Program, Vanderbilt University, Nashville, TN, USA
8. Evolutionary Studies Initiative, Vanderbilt University, Nashville, TN, USA

Table S1. *E. coli* IC residue compositions at cutoff = 0.95.

	*ec*DHFR residues
IC1	13,23,25,27,32,39,55,63,71,107,133,153
IC2	7,14,15,31,35,42,43,44,46,49,53,54,57,59,94,95,96,100,113,122,126
IC3	21,22,24,50,52,64,81,121,
IC4	5,6,11,18,40,45,47,51,92,111,125,

Table S2. Within variant p-values from Mann-Whitney U tests of dynamic correlations for *E. coli* DHFR.

**Comparison Category**	**3QL3**	**3QLO**

IC1 vs No IC	0.44296	0.02679
IC2 vs No IC	1.79839e-08	0.00922
IC3 vs No IC	0.04803	0.10210
IC4 vs No IC	0.04503	0.21941
IC1 vs Not in Same IC	0.61656	0.02452
IC2 vs Not in Same IC	1.29761e-07	0.00663
IC3 vs Not in Same IC	0.07079	0.10267
IC4 vs Not in Same IC	0.07716	0.25263
Any IC vs No IC	3.45887e-18	9.41947e-05
Not in Same IC vs No IC	0.09708	0.67440

Table S3. Across variant p-values from Mann-Whitney U tests of dynamic correlations for *E. coli* DHFR.

Comparison Category	Comparison
IC1 (3QL3) vs IC1 (3QL0)	0.00283
IC2 (3QL3) vs IC2 (3QL0)	6.17574e-12

IC3 (3QL3) vs IC3 (3QL0)	0.03087
IC4 (3QL3) vs IC4 (3QL0)	2.43828e-06
Any IC (3QL3) vs Any IC (3QL0)	1.09078e-70
No IC (3QL3) vs No IC (3QL0)	7.79406e-193
Not in Same IC (3QL3) vs Not in Same IC (3QL0)	0.00000e+00

Table S4. Human DHFR IC residue compositions at cutoff = 0.95.

	Human DHFR residues
IC1	15,26,28,30,35,47,49,68,76,85,128,156,179
IC2	9,16,17,34,38,52,53,54,56,59,66,67,70,72,115,116,117,121,136,145,149
IC3	23,24,27,60,65,77,96,144
IC4	7,8,13,20,50,55,57,61,113,134,148

TableS5. Proteins for which the Met20 Pro-Pro motif was present in our original alignment and which did not possess either the Gly20 mutation or the exact 61-PEKN-65 mutation. PFAM protein accession numbers are in the first column with common or scientific names available in the second column.

PFAM accession	Name	Gly20	Met20 Loop motif	P61-N65
R7UI73	*Capitella teleta* (Polychaete worm)	C	PP	GEEE
U6GUT1	*Eimeria acervulina* (Coccidian parasite)	N	PP	empty
S7NYA8	brandts bat	G	PP	PKKN
G1QES9	little brown bat	G	PP	PKKN
L5KJI5	black flying fox	G	PP	PKKN
S7NHW6	brandts bat	G	PP	PKKN
M1VWK3	*claviceps purpurea* (ergot)	G	PP	PPSF
A0A2Z5U771	*Rhinolophus gammaherpesvirus 1* (from greater horshoe bat)	G	PP	PTKS
A0A3L8S6E8	*Chloebia gouldiae* (Gouldian finch)	G	PP	PEKS

U3IIA3	*Anas platyrhynchos platyrhynchos* (Northern mallard)	G	PP	PEKH

Table S6. Within variant p-values from Mann-Whitney U tests of dynamic correlations for the human variant (4M6K). The “original MSA” represents p-values for the first conserved network identified in human DHFR and the “biased MSA” column represents.

Comparison Całegory	4M6K Original MSA	4M6K Biased MSA
IC1 vs No IC	0.67861	0.06398
IC2 vs No IC	0.99219	1.79859e-04
IC3 vs No IC	0.89580	0.00937
IC4 vs No IC	0.54960	0.57547
IC1 vs Not in Same IC	0.99119	0.12690
IC2 vs Not in Same IC	0.58259	5.90955e-05
IC3 vs Not in Same IC	0.89361	0.07172
IC4 vs Not in Same IC	0.75002	0.40275
Any IC vs No IC	0.23658	2.68604e-05

Not in Same IC vs No IC	0.01681	0.00363

Table S7. New Human DHFR IC residue compositions at cutoff = 0.95. SCA was performed on the original MSA which was created by removing sequences with less than 40% sequence similarity to *h*DHFR.

	New Human DHFR residues
IC1	8,83,86,87,110,128,134,135,148,156
IC2	50,52,54,55,56,60,65,70,72,182
IC3	7,9,15,16,17,20,22,23,24,27,34,38,48,53,61,66,67,116,117,121,136,142,144,145,147,149
IC4	30,69,113,138,174,175,177
IC5	31,57,59,63,64,76,115
IC6	11,13,37,114,118,125,129,132,146,170,171,172,173,179,181,183

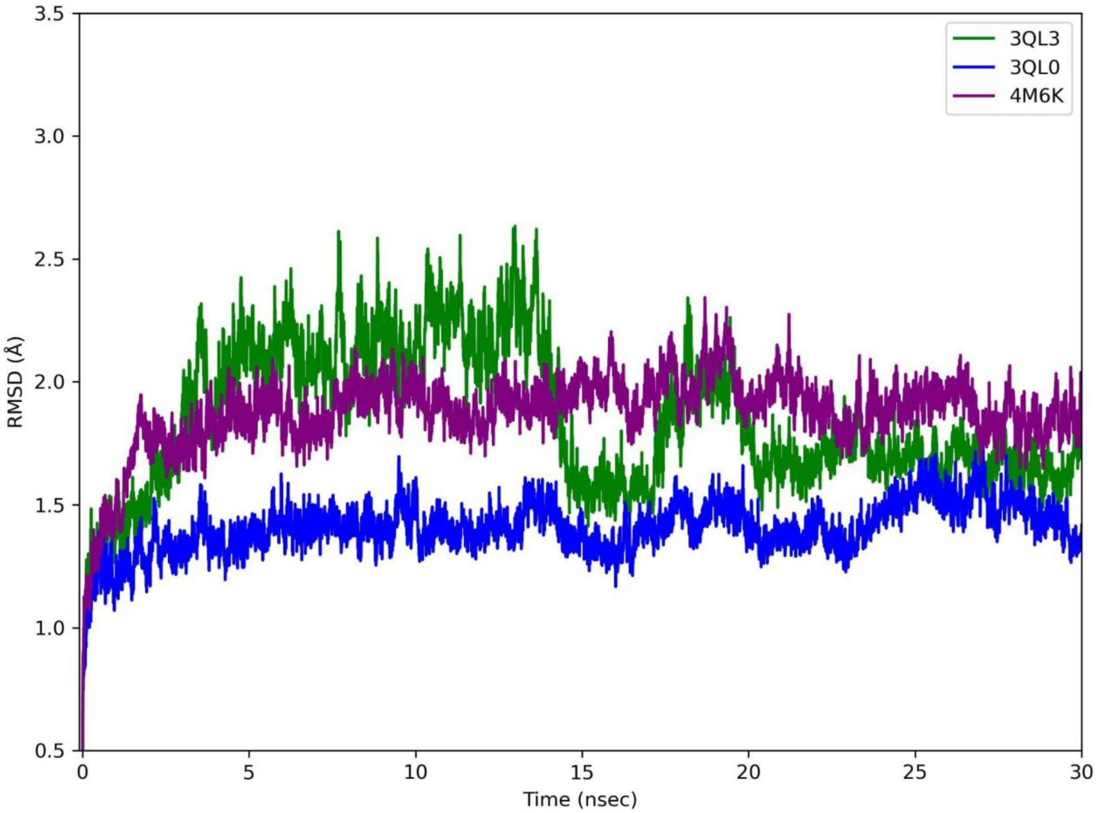

Figure S1. The root-mean squared deviation (RMSD) of human DHFR (4M6K, red), wild-type *ec*DHFR (3QL3, green), and mutant *ec*DHFR (3QL0, blue) with respect to their initial structures over the duration of 30 ns simulation.

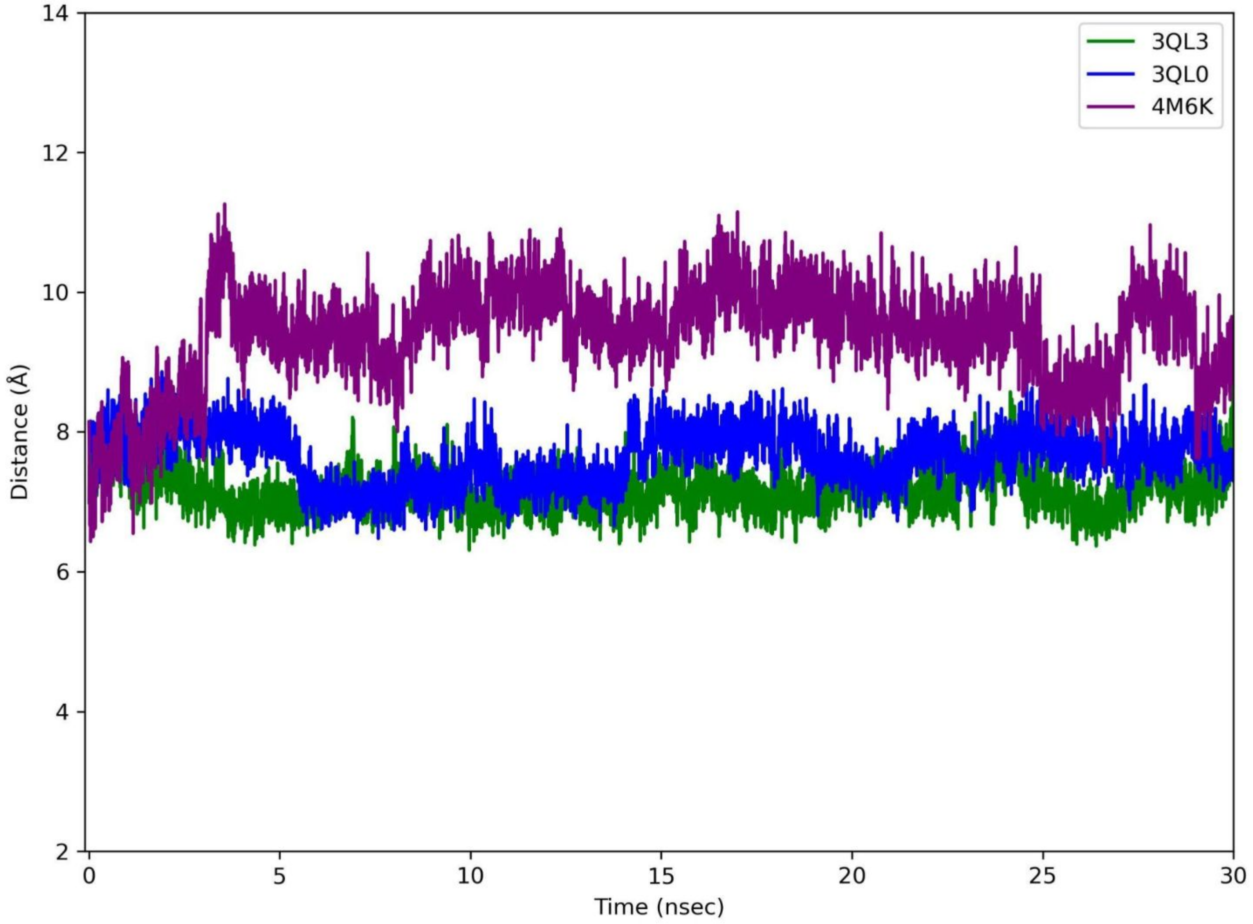

Figure S2. The distance between the folate ligand and a residue in the active site of wild-type *ec*DHFR (green), mutant *ec*DHFR (blue), and *h*DHFR (red) over the course of 30ns simulation.

Simulation stability.

Figure S1 monitors the RMSD of the proteins with respect to the first frame of the trajectory over the simulation time. Simulations of the mutant *ec*DHFR and *h*DHFR are shown to be reasonably stable, with fluctuations gradually increasing then stabilizing at approximately 1.4 and 1.9 Å, respectively, starting at around 5 ns. On the other hand, the wild-type *ec*DHFR exhibited larger fluctuations. Further analysis suggests that these fluctuations reflect the structural flexibility especially the Met20 loop and its hinge motion of the wild-type *ec*DHFR in comparison to the other DHFR simulations (**Figures S3 and S5**). The wild-type *ec*DHFR sampled structures closer to the open state where the RMSD values were greater than ~1.8Å (at around 5-13ns and 18-20ns) and resemble the closed state below ~1.8Å (at around 14-18ns) **(Figure S6).** Distance between the folate ligand and a residue in the DHFR active site (positions K32 in *ec*DHFR and Q35 in *h*DHFR) from center of mass was also calculated to monitor the stability of the ligand-amino acid interaction during the simulation. **Figure S2** shows this distance averages at around 7.2 to 7.6 Å for wild-type and mutant *ec*DHFR, respectively, and around 9.3 Å for *h*DHFR.

Fluctuations are within a range of 2 Å, suggesting relative stability of this interaction throughout the simulation.

A

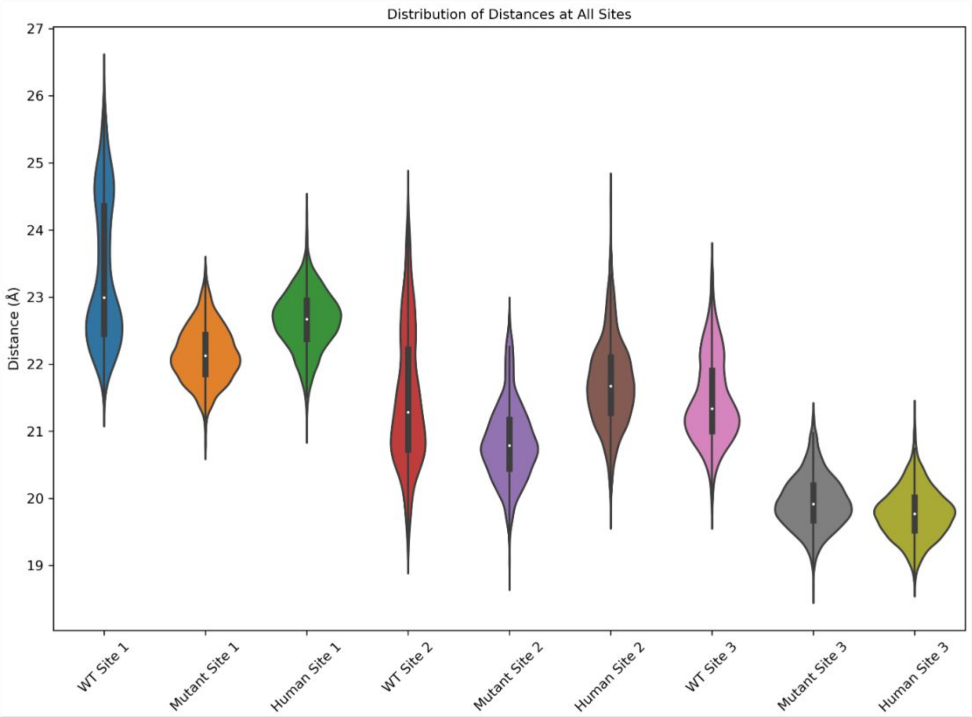

B

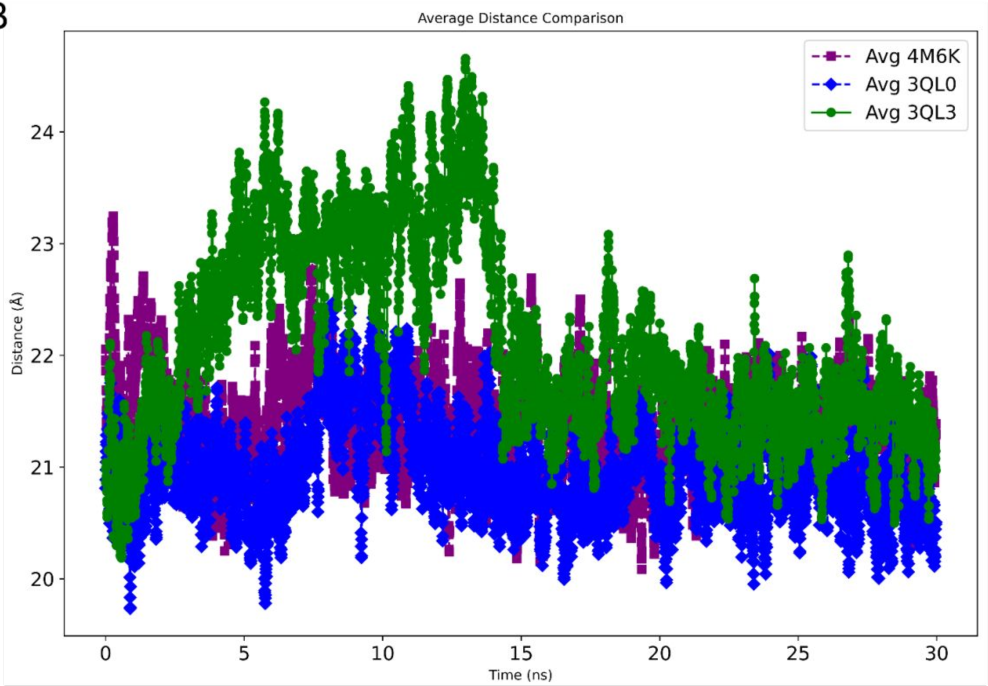

Figure S3. Distribution of hinge distances at three different sites in *ec*DHFR and *h*DHFR **(A)**. Wild-type hinge distance were averaged between positions W22-P53, N23-P53 and L24-P53 (Site 1-3, respectively) while mutant hinge distance is the average of the distance between positions W22-P54, P23-P54 and L25-P54 (Site 1-3, respectively). Human DHFR hinge distance was averaged between positions W24-P66, P25-P66, and L27-P66 (Site 1-3, respectively). Hinge distance average of the three sites for wild-type (green) and mutant *ec*DHFR (blue) and *h*DHFR (purple) **(B).** All measurements were taken at the alpha carbon and measured throughout the 30ns simulation.

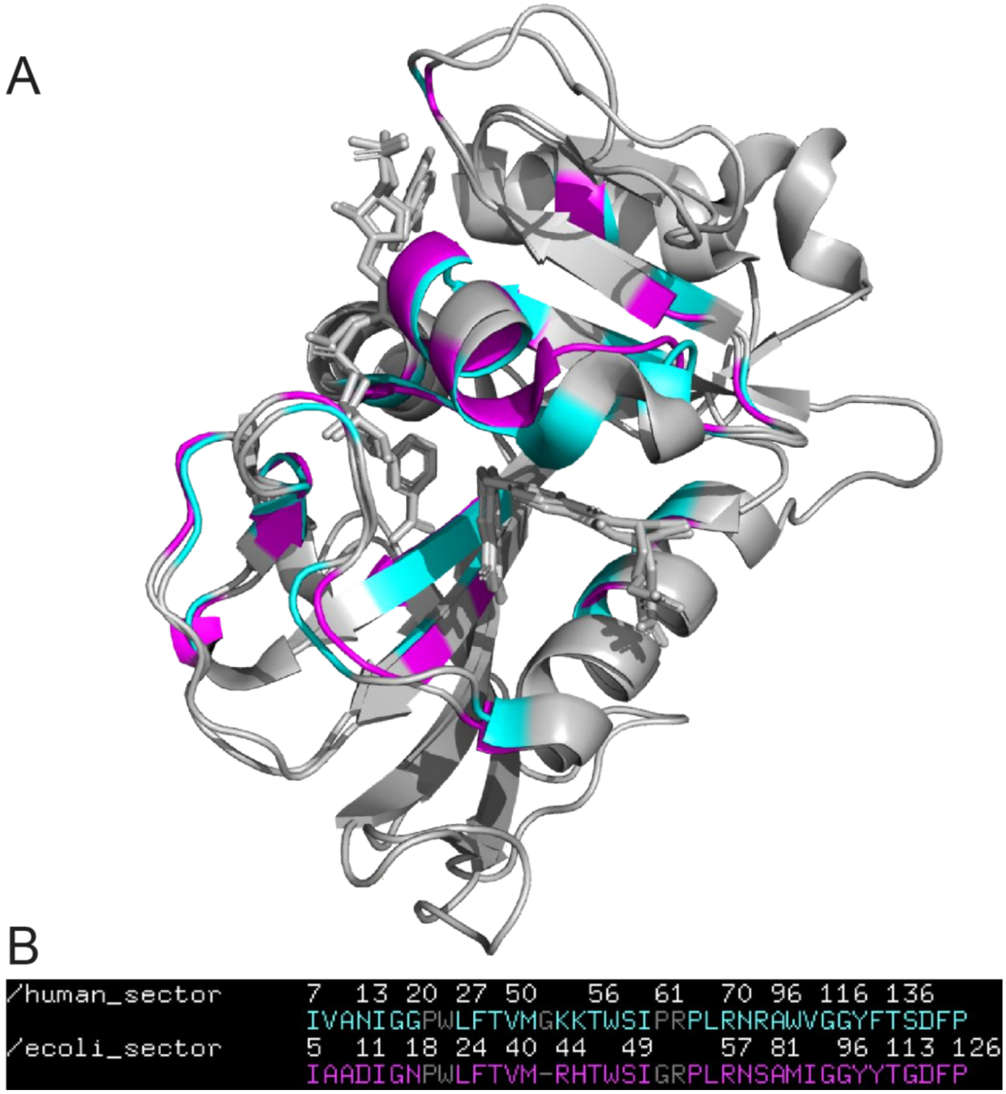

Figure S4. Overlaid PDB structures for human (PDB:4M6K) and *E. coli* (PDB:3QL3) DHFR **(A)** along with the structure-based alignment of their sectors (ICs 2-4) **(B)**. The RMSD for the structure-based alignment was 0.853Å after 5 cycles with a total of 42 rejected atoms. Numbering is only accurate for the amino acid over which it appears and is meant as a reference for the others.

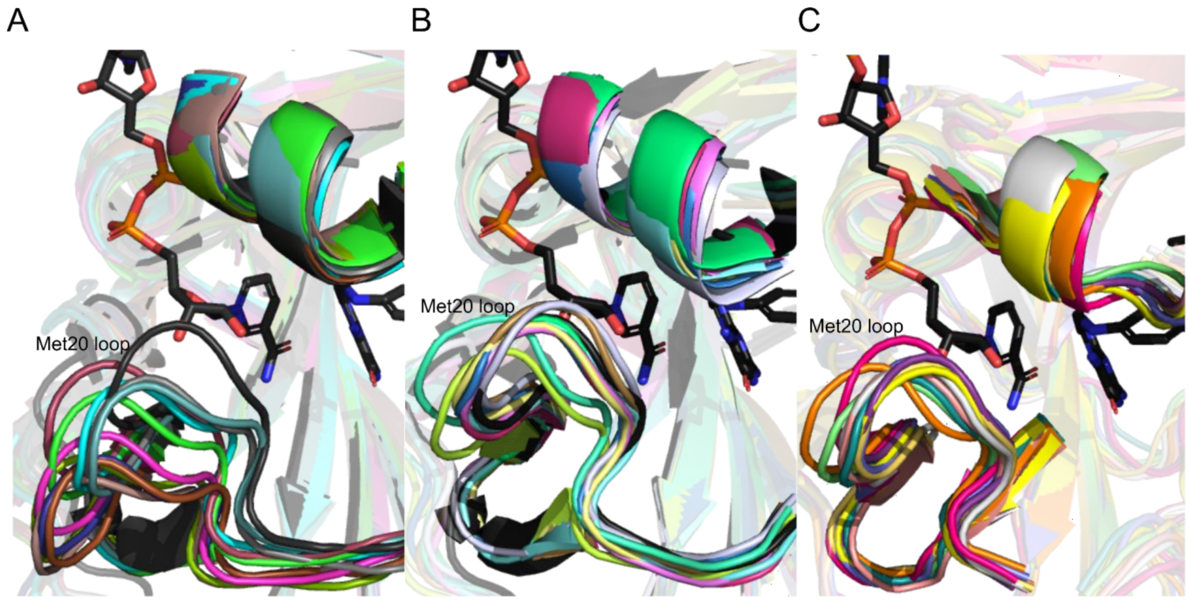

Figure S5. Top ten representative conformations throughout the 30ns simulation adopted by wild-type *ec*DHFR **(A)**, mutant *ec*DHFR (**B**), and human DHFR, highlighting the Met20 loop and alpha-helix containing *e*Ser49 and *h*Ser59. Structures were obtained by *cpptraj* K-means algorithm clustering using the options "cluster c1 kmeans clusters 10 randompoint maxit 500 rms :1-159@C,N,O,CA,CB&!@H= sieve 10 random". Structures colored in black represent the deposited PDB structures 3QL3, 3QL0, and 4M6K, respectively.

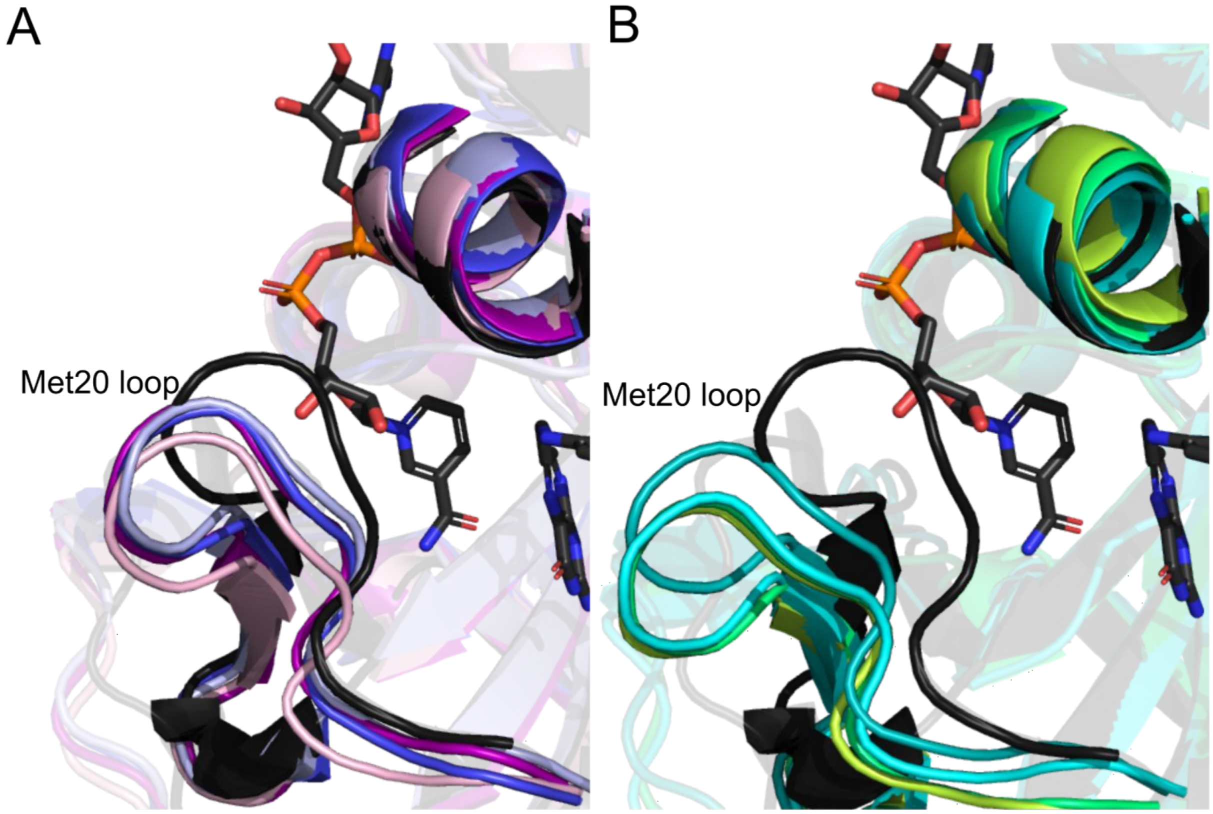

Figure S6. Conformations of the wild-type *ec*DHFR closely representing the more native state **(A)** and open state **(B)** of the Met20 loop during the simulation. Structures were obtained by *cpptraj* K-means algorithm clustering using the options “cluster c1 kmeans clusters 2 randompoint maxit 500 rms :1-159@C,N,O,CA,CB&!@H= sieve 10 random” at time points where the RMSD fluctuations **(Figure S1)** were above **(A)** (at around 5-13ns and 18-20ns) or below ~1.8Å (at around 14-18ns) **(B)**. Structures colored in black represent the deposited 3QL3 PDB structure.

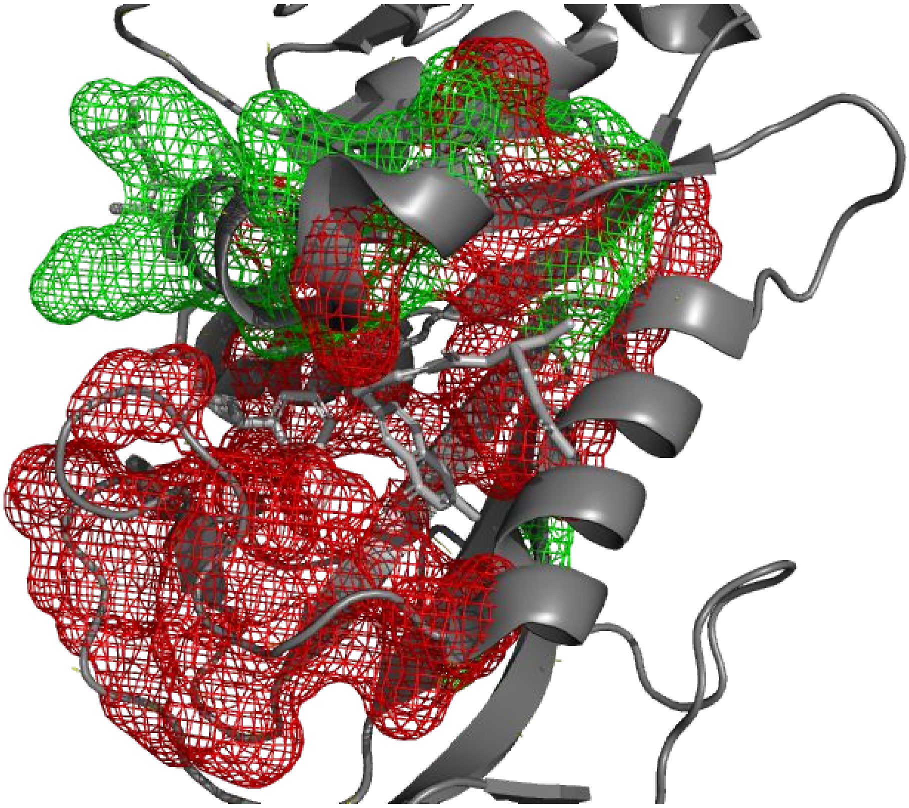

Figure S7 IC2 (green) and IC3 (red) identified by removing all sequences with less than 40% identity to human DHFR and re-computing the SCA matrix. ICs are mapped onto human DHFR (PDB: 4M6K).
